# Differential metabolism of choline supplements in adult volunteers

**DOI:** 10.1007/s00394-021-02637-6

**Published:** 2021-07-21

**Authors:** Katrin A. Böckmann, Axel R. Franz, Michaela Minarski, Anna Shunova, Christian A. Maiwald, Julian Schwarz, Maximilian Gross, Christian F. Poets, Wolfgang Bernhard

**Affiliations:** 1 Department of Neonatology, Faculty of Medicine, Eberhard-Karls-University, Calwer Straße 7, 72076 Tübingen, Germany; 2 Center for Pediatric Clinical Studies, Faculty of Medicine, Eberhard-Karls-University, Calwer Straße 7, 72076 Tübingen, Germany

**Keywords:** Choline, Glycerophosphocholine, Supplementation, Trimethylamine oxide

## Abstract

**Background:**

Adequate intake of choline is essential for growth and homeostasis, but its supply does often not meet requirements. Choline deficiency decreases phosphatidylcholine (PC) and betaine synthesis, resulting in organ pathology, especially of liver, lung, and brain. This is of particular clinical importance in preterm infants and cystic fibrosis patients. We compared four different choline supplements for their impact on plasma concentration and kinetics of choline, betaine as a methyl donor and trimethylamine oxide (TMAO) as a marker of bacterial degradation prior to absorption.

**Methods:**

Prospective randomized cross-over study (1/2020–4/2020) in six healthy adult men. Participants received a single dose of 550 mg/d choline equivalent in the form of choline chloride, choline bitartrate, α-glycerophosphocholine (GPC), and egg-PC in randomized sequence at least 1 week apart. Blood was taken from *t* = − 0.1–6 h after supplement intake. Choline, betaine, TMAO, and total PC concentrations were analyzed by tandem mass spectrometry. Results are shown as medians and interquartile range.

**Results:**

There was no difference in the AUC of choline plasma concentrations after intake of the different supplements. Individual plasma kinetics of choline and betaine differed and concentrations peaked latest for PC (at ≈3 h). All supplements similarly increased plasma betaine. All water-soluble supplements rapidly increased TMAO, whereas egg-PC did not.

**Conclusion:**

All supplements tested rapidly increased choline and betaine levels to a similar extent, with egg-PC showing the latest peak. Assuming that TMAO may have undesirable effects, egg-PC might be best suited for choline supplementation in adults.

**Study registration:**

This study was registered at “Deutsches Register Klinischer Studien” (DRKS) (German Register for Clinical Studies), 17.01.2020, DRKS00020454.

**Supplementary Information:**

The online version contains supplementary material available at 10.1007/s00394-021-02637-6.

## Introduction

Choline is an essential nutrient, and its supply is regarded as critical in parts of the general population and certain medical conditions [1–3]. It is required for the formation of phosphatidylcholine (PC) and sphingomyelin (SPH), two phospholipids with tightly regulated concentrations in the cell membranes of all tissues and many secretions (bile, lipoproteins, surfactant). Choline is also required for the synthesis of acetylcholine (ACh), a neurotransmitter fundamental to fetal synaptogenesis and the development and function of the brain [4–6]. PC is, moreover, essential to sphingolipid metabolism, as it is substrate of sphingomyelin synthase (EC 2.7.8.27) for the formation of SPH from (pro-) apoptotic ceramides [7–9]. A substantial part of choline is oxidized to betaine, being an osmolyte important to kidney function, and having a rapid turnover in the liver where it is primarily used for the regeneration of methionine from homocysteine. Via S-adenosyl methionine, it serves a plethora of important methylation processes from creatine synthesis to epigenetic control via DNA/histone methylation [10].

Adequate intake of choline must be ensured because endogenous choline synthesis does not meet demand. Therefore, the US-Food and Drug Administration defined choline as an essential nutrient in 1998, and both the National Academy of Medicine of the USA (NAM) and the European Food Safety Authority (EFSA) defined adequate intake (AI) values. The AI of healthy adult males by the NAM is considered to be 550 mg/d (= ~ 8 mg/kg/d). AI varies with age, sex, life conditions (pregnancy, breastfeeding), and genetic polymorphisms [11–13].

Sufficient choline supply is exceptionally important for preterm infants. During pregnancy, choline is actively transported via the placenta to the fetus [14]. In preterm infants, we showed that plasma levels fall to 50% of fetal values after birth [15]. Such decrease, developing physiologically after term delivery, occurs during a period of extremely rapid growth, when high choline levels would be physiological. Another group with high choline requirements is cystic fibrosis (CF) patients. Through exocrine pancreas insufficiency, these patients have a deficiency of hydrogen carbonate secretion and pancreatic phospholipase A2 IB (sPLA2IB), which are required for the cleavage of PC to lyso-PC and fatty acids, which is essential for the salvage of its choline moiety via the enterohepatic cycle. Typically, choline deficiency leads to mobilization of choline from other organs like the lung to maintain liver function [16, 17]. There is a correlation between choline status and lung and liver function in CF patients, which may similarly apply to preterm infants [16].

Whereas choline supplementation appears mandatory for the above-mentioned medical conditions, there have also been concerns that choline (over-) supplementation may have adverse effects: if choline is not fully absorbed in the small intestine, trimethylamine (TMA) is formed as a bacterial degradation product in the colon. Moreover, small intestinal bacterial overgrowth (SIBO) may result in the formation of TMA from choline prior to its absorption within the small intestine, impairing choline bioavailability. In adults and term infants, the liver oxidizes trimethylamine to trimethylamine oxide (TMAO) by flavin-containing monooxygenase 3 (FMO3), which is associated with cardiovascular events [3, 18–20]. We were interested whether there are differences in the generation of TMAO from choline salts compared to its organic derivatives.

It was shown before that levels of free choline were similar after the intake of phosphatidylcholine and choline bitartrate but TMAO showed higher levels after the intake of choline bitartrate [21]. Others demonstrated that plasma choline levels were higher after the intake of phosphocholine compared to levels after the intake of choline bitartrate [22].

Because of different mechanisms of choline accretion from different choline sources, we aimed to systematically investigate the kinetics of plasma choline and its metabolites following administration of 4 different choline supplements, namely choline chloride, choline bitartrate, GPC, and egg-PC. The results of this trial are a basis to define an optimal enteral choline supplement for adults with choline deficiency, and may help to optimize choline supplementation of preterm infants.

## Methods

This is a randomized cross-over study on six adult men, carried out at Tuebingen University Hospital, Germany. Test persons were recruited from January 2020 to April 2020. The Institutional Review Board (project number 322/2019BO1) approved the protocol, and written informed consent was obtained prior to enrolment.

### Inclusion criteria

Test persons had to be male and at least 18-years-old.

### Exclusion criteria

Exclusion criteria included alcohol abuse, acute illness, chronic diseases (like diabetes, metabolic syndrome, thyroid diseases, pancreas insufficiency), intake of choline-containing nutritional supplements, or missing consent.

### Supplements

Choline chloride (CAS: 67-48-1), choline bitartrate (CAS: 87-67-2) and α-glycerophosphocholine (GPC; CAS: 28319-77-9) have GRAS (Generally Recognized As Safe) status. Egg-phosphatidylcholine (egg-PC; CAS: 8002-43-5) is a natural choline source in human nutrition. These substances were received via the University Pharmacy Department, and were from Merck (Darmstadt, Germany) (choline chloride, choline bitartrate) or Lipoid (Ludwigshafen, Germany) (GPC, egg-PC). Aliquoted choline supplements were provided as dry substances for oral use in 100 ml sterile brown glass bottles by Rainfarn-Apotheke (Munich, Germany).

### Study schedule

The test subjects had to drink the adequate intake (AI) of choline according to NAM (550 mg/d) [12] in the form of:

- 740 mg choline chloride
- 1336 mg choline bitartrate
- 1358 mg GPC
- 4018 mg egg-PC.

Subjects took each of the four supplements in randomized sequence with interspersed wash-out periods of at least 1 week. Substances were dissolved (choline chloride, choline bitartrate, GPC) or suspended (egg-PC) in 50 ml sterile water before use and diluted in 250 ml apple spritzer. The test started in the morning after overnight fasting, and the supplement was served together with a butter pretzel (choline/butter pretzel: ≈2 mg choline in 10 g butter + ≈30 mg choline in 94 g pretzel). To exclude an effect of the test meal on target parameters, test persons 1 and 2 ingested 250 ml apple spritzer together with a butter pretzel without test substance. Blood (2.7 ml EDTA) was taken before intake (− 0.1 h) and at 0.5, 1, 1.5, 2, 3, 4, 5, 6, and 24 h after intake. Test persons fasted until the 6 h-blood sample was collected. Collected blood was immediately centrifuged at 1000 × *g* at room temperature for 10 min. Plasma was separated and stored at − 80 °C until analysis.

### Chemical analysis

Plasma samples were processed with established standard procedures. In brief, plasma was spiked with internal standard (D_4_-choline chloride, diarachinoyl-PC [PC20:0/20:0]), and was extracted with chloroform:methanol according to Bligh and Dyer. The upper water:methanol phase, containing water-soluble choline and its metabolites, was separated from the lipid-containing chloroform phase [15, 16, 23, 24]. Equipment for analysis comprised a TSQ Quantum Discovery MAX tandem mass spectrometer, a Finnigan Surveyor Autosampler Plus, and a Finnigan Surveyor MS Pump Plus (Thermo Fisher Scientific, Dreieich, Germany).

Choline, D_4_-choline, betaine, dimethylglycine, phosphocholine, GPC, and TMAO were separated on a Polaris Si-A® column (2 × 100 mm inner diameter; 3.0 µm particle size) at 40 °C. Elution was performed at 0.5 ml/min with solvent A (methanol:water:formic acid; 95:5:1; v/v) and B (water:formic acid, 99: 1; v/v): Gradient conditions were 100% A (0–2 min) → 50% B (2–4 min) → 100% A (4–5 min) → 100% A (5–7 min), and components were analyzed at positive ionization.

PC, lyso-PC, and SPH were separated on a Polaris Si-A (2 × 150 mm i.d.; 2 µm; Agilent Technologies) with chloroform:methanol: 300 mM ammonium acetate (60:38:2%, v/v) as the mobile phase. Phosphorylcholine (mass/charge [m/z] =  + 184) served as the diagnostic fragment [15, 16, 23, 24].

### Statistics

Differences between choline derivatives regarding plasma kinetics or metabolic rates were not known. Calculation of case numbers was, therefore, based on plasma choline levels of patients with and without choline supplementation. In adults (*n* = 10), the average (± standard deviation) plasma choline level was 5.2 ± 1.6 µmol/l without and 13.7 ± 5.3 µmol/l with supplementation (2200 mg/d, corresponding to 34 mg/kg/d at a mean weight of 65 kg) [26]. Assuming normal distribution of the data, a similar effect size and *α* ≤ 0.05, six individuals had to be analyzed to verify this clinically relevant impact on choline concentration with a power of 80%.

The primary outcome was defined as the area under the choline concentration curve (0–24 h), pre-defined secondary outcome variables were the change of plasma concentration of choline and its metabolites from baseline (defined as slope of concentration change until peak concentration and peak value of the plasma concentration change). Post hoc, the area under curve for choline plasma concentration (0–6 h) and time-point of peak concentrations of choline and betaine were added as outcome parameter.

Statistical and graphical analyses were done using JMP 14 (SAS Institute GmbH, Germany), GraphPad Prism Version 8.4.0 (GraphPad Software, San Diego, California, USA), and Excel 2010 (Microsoft Corporation, USA). Normal distribution was tested with Shapiro–Wilk test. Because many parameters were not normally distributed they are shown as median with 25th‰ and 75th‰ and non-parametric tests were used. Additionally, individual data points are shown. Area under the curve was calculated with Excel (trapezoidal rule).

AUC of choline and betaine following supplementation were globally compared by Friedman test (using GraphPad), and subsequently were compared between individual groups by Wilcoxon signed-rank (using JMP). This procedure was also used to compare the time to peak and slope to peak of choline concentrations. We applied a significance level of *p* = 0.05. Correction for multiple testing was not applied in this exploratory analysis.

## Results

The study was carried out in the Neonatal Department of Tuebingen University Children’s Hospital from January to April 2020. Demographic data of the study participants are shown in Table 1. Shapiro–Wilk test showed mostly normally distributed values for choline, whereas for betaine and TMAO values were not normally distributed. 24 h after supplementation, plasma concentrations had returned to baseline (0 h) values (Table 2). There was no difference between the area under the curve (AUC) at 0–24 h and 0–6 h for choline plasma concentrations after administration of the four different supplements (Table 3 and Supplemental Table 1). The AUC (0–24 h and 0–6 h) of betaine concentrations had the highest values for egg-PC and lowest for choline chloride (Table 3 and Supplemental Table 1). The choline/betaine ratios were similar for all supplements (Supplemental Table s2).

**Table 1:** Demographic data of participants

Number	6
Weight (kg)	74.5 (71–90.9) [68.0–99.5]
Height (cm)	184 (176–187) [175–191]
Age (years)	37 (35–56) [31–64]

Data are shown as median, (25th‰–75th‰) and [range]

**Table 2:** Choline, betaine, and TMAO concentrations before and 24 h after intake of choline supplements

	–	Choline chloride	Choline bitartrate	αGPC	Egg-PC
	0 h	24 h			
	*n* = 24	*n* = 6	*n* = 6	*n* = 6	*n* = 6
	(µmol/l)				
Choline	8.6 (7.55–9.83)	8.64 (5.95–8.85)	8.3 (6.78–9.26)	8.37 (7.35–10.38)	8.12 (6.2–9.05)
Betaine	20.4 (18.2–31.4)	20.2 (19.6–29.8)	23.5 (21.8–38.9)	23.8 (22.6–31.7)	24.0 (21.9–28.0)
TMAO	1.99 (1.22–3.07)	3.75 (3.29–6.52)	2.25 (2.08–4.19)	2.64 (1.75–6.05)	1.68 (1.24–4.01)

Data are shown as median (25th‰–75th‰). Concentrations are shown in µmol/l

*PC* phosphatidylcholine, *TMAO* trimethylamine oxide, *αGPC* alpha-glycerophosphocholine

**Table 3:** Area under the curve for choline and betaine plasma concentrations after intake of choline supplements

	Choline chloride	Choline bitartrate	αGPC	Egg-PC
AUC of plasma choline concentration (0–24 h) µmol/l × h	235.2 (206.5–261.1)	233.5 (183.3–274.4)	244.9 (185.3–268.6)	244.3 (189.2–289.7)
AUC of plasma choline concentration (0–6 h) µmol/l × h	63.4 (54.9–72.4)	66.4 (48.9–75.5)	61.6 (47.4–71.9)	67.9 (56.2–82.6)
AUC of plasma betaine concentration (0–24 h) µmol/l × h	537.3 (499.0–871.2)	625.0 (570.8–993.9)	643.4 (552.9–923.3)	665.4 (580.9–840.9)
AUC of plasma betaine concentration (0–6 h) µmol/l × h	143.9 (126.9–208.3)	156.3 (142.5–211.6)	150.5 (129.6–229.8)	160.8 (144.9–197.7)

Data are shown as median (25th‰–75th‰). Data of all subjects were included

*AUC* area under the curve, *h* hour, *PC* phosphatidylcholine, *TMAO* Trimethylamine oxide, *αGPC* alpha-glycerophosphocholine

**Fig. 1:**
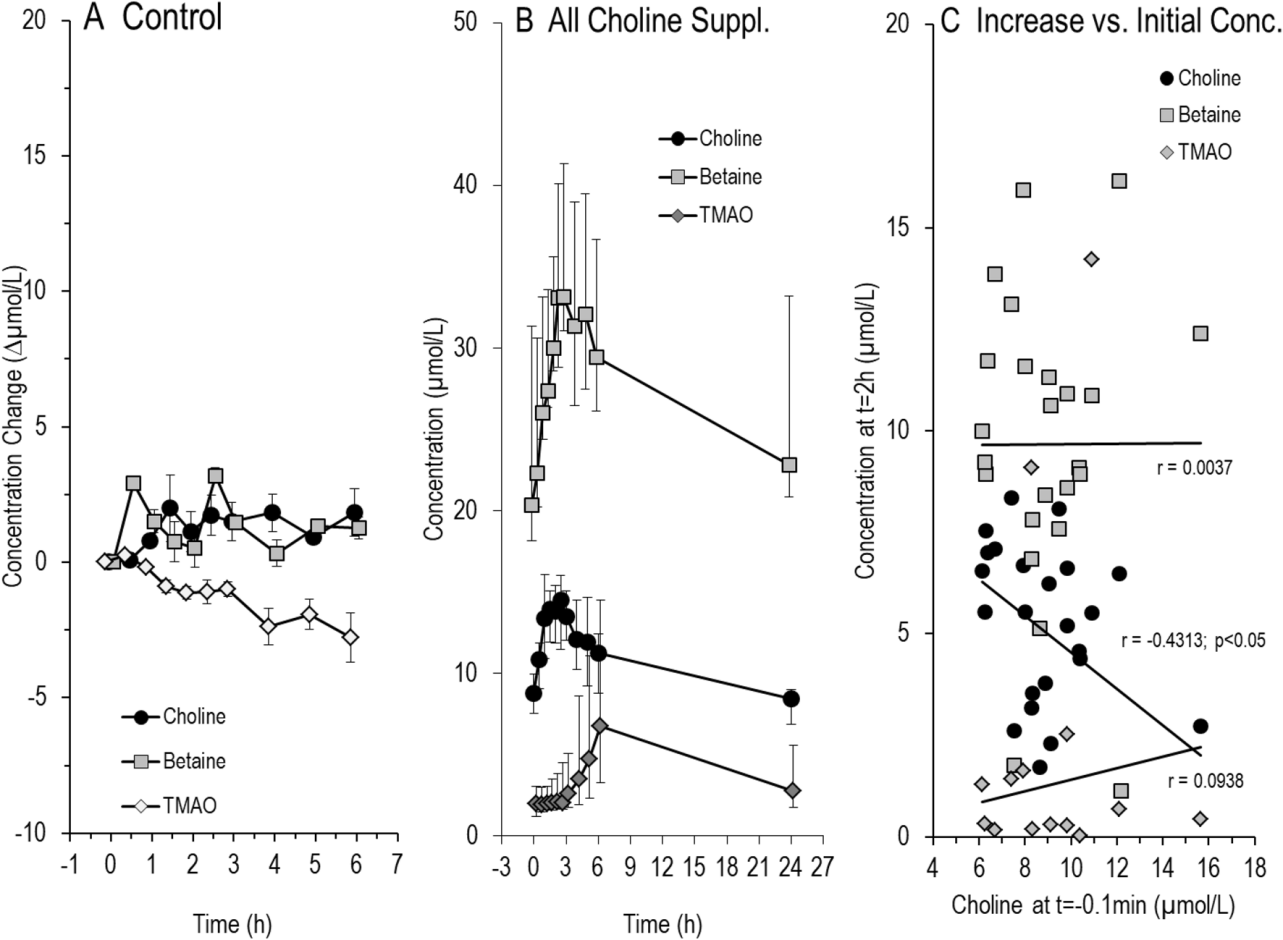
Time course of choline, betaine, and TMAO in control and all supplementation experiments. Data show that **A** a butter pretzel and 250 mL apple spritzer followed by 6 h fasting only slightly increased choline and betaine and did not increase TMAO concentrations. **B** demonstrates the kinetics of choline, betaine, and TMAO concentration over all supplements. Data are indicated as medians and interquartile ranges. **C** shows the relation of initial plasma choline concentration on choline, betaine, and TMAO levels 2 h after supplement ingestion

Figure 1A demonstrates that the test meal without choline supplement, followed by 6 h fasting, moderately increased choline and betaine, whereas trimethylamine oxide (TMAO) decreased. By contrast, summing up all supplementation experiments, there was a rapid increase in plasma choline from 8.75 (7.52–9.96) µmol/l to 14.51 (11.48–16.04) µmol/l, and in betaine from 20.4 (18.2–31.4) µmol/l to 33.1 (28.8–40.1) µmol/l (Fig. 1B). Thereafter, median plasma concentrations of choline and betaine decreased, returning to baseline at 24 h (Table 2), those of trimethylamine oxide (TMAO) steadily increased from 2.0 (1.22–3.07) µmol/l at *t* = 0 h to 6.73 (3.25–14.52) µmol/l at 6 h (Fig. 1B), and thereafter decreased to baseline after 24 h as well (Table 2). Notably, the extent of increase in plasma choline inversely correlated with its initial concentration, whereas this did not apply to betaine and TMAO (Fig. 1C).

**Fig. 2:**
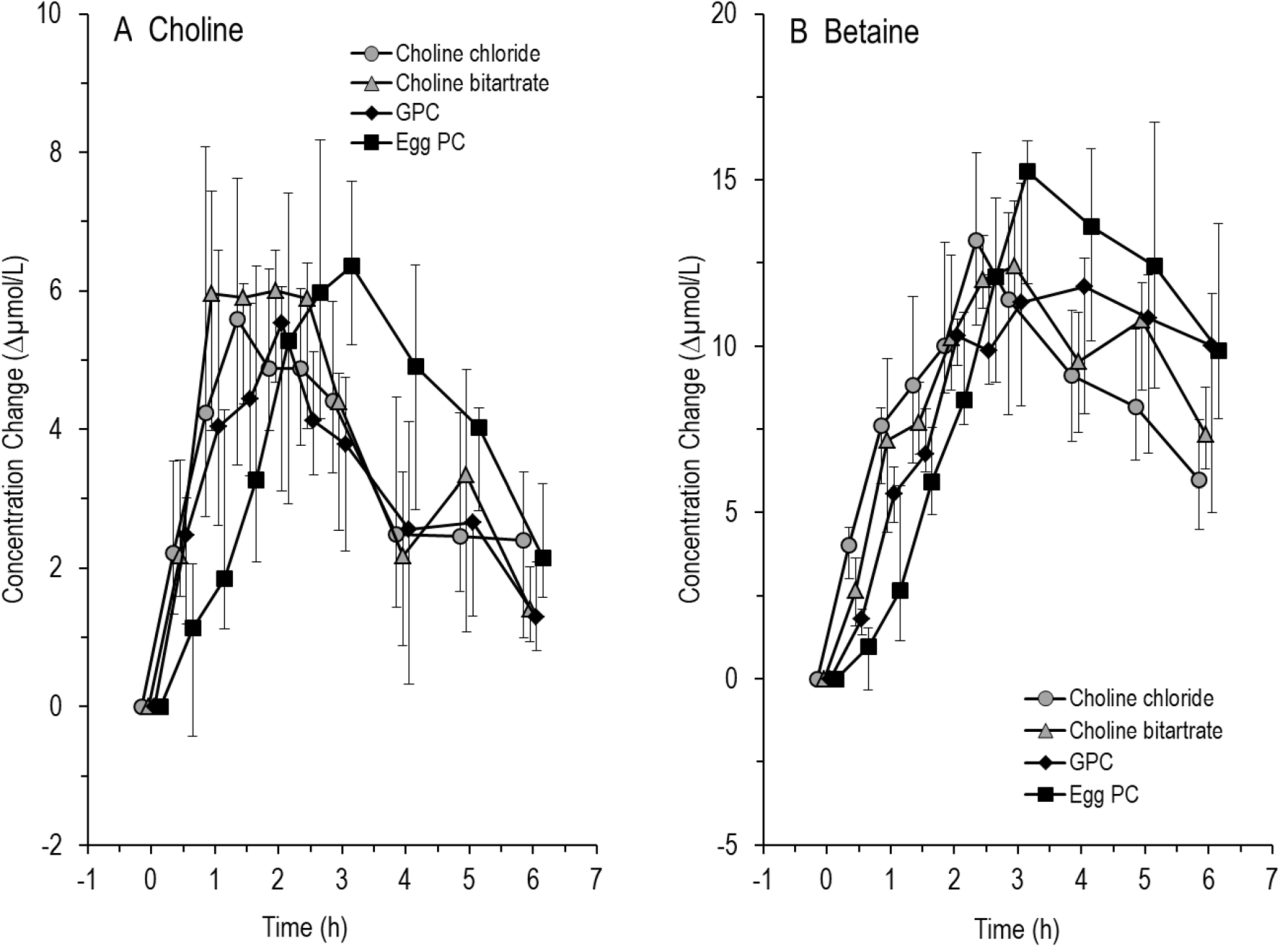
Time course of choline (**A**) and betaine (**B**) in response to different choline components (*N* = 6 for each group). Choline components were given orally as a single dose of 550 mg choline equivalent together with a test meal. Data are medians and interquartile ranges

Table 4 shows the time-point of the peaks for choline and betaine concentrations after intake of the four supplements. Time to peak was longest for egg-PC with a significant difference in time to peak between egg-PC and GPC (*p* = 0.03) and choline bitartrate (*p* = 0.06). The difference to the other groups was not significant (*p* > 0.05) (Supplemental Table 1). The slope to peak was highest for choline bitartrate (Table 4). There was no significant difference in the slope to peak between the supplements (Friedman *p* = 0.26) (Supplemental Table 1). Figure 2A and B demonstrates the effects of individual choline supplements on the extent and kinetics of plasma choline and betaine concentration, respectively. The slope of increase in plasma choline was highest for choline bitartrate, with peak concentrations reached at 1–2 h, whereas the slope following egg-PC was lower (Table 4), and peak concentration was reached at approximately 3 h (Fig. 2A). Increases in peak concentrations were similar following all components at about + 6 µmol/l.

**Table 4:** Time to peak choline and betaine concentrations and slope to peak choline concentration after the intake of choline supplements

	Choline chloride	Choline bitartrate	αGPC	Egg-PC
Time to choline concentration peak (h)	2.25 (0.88–3.5)	1.75 (1.0–2.13)	1.75 (1.38–2.5)	2.75 (2.0–3.5)
Time to betaine concentration peak (h)	2.5 (2.25–4.25)	2.75 (2.0–3.5)	3.0 (2.38–4.25)	4.0 (3.0–5.0)
Slope to peak choline concentration (Δ choline concentration/time to peak)	2.23 (1.72–13.06)	4.76 (1.99–8.71)	2.94 (2.07–5.72)	2.49 (1.59–3.54)

Data are shown as median (25th‰–75th‰)

*h* hours, *PC* phosphatidylcholine, *TMAO* Trimethylamine oxide, *αGPC* alpha-glycerophosphocholine

Similar kinetics applied to betaine (Fig. 2B), with peak concentration being reached at 4 h in response to egg-PC, and earlier for all other compounds. Notably, increases in plasma betaine concentrations surmounted those of choline, and were at 12–15 µmol/l.

Plasma GPC remained low throughout [0.82 (0.66–1.06) µmol/l], and no supplement changed its plasma concentrations nor that of phosphocholine, dimethylglycine, methionine, carnitine, PC, lyso-PC or SPH (not shown).

**Fig. 3:**
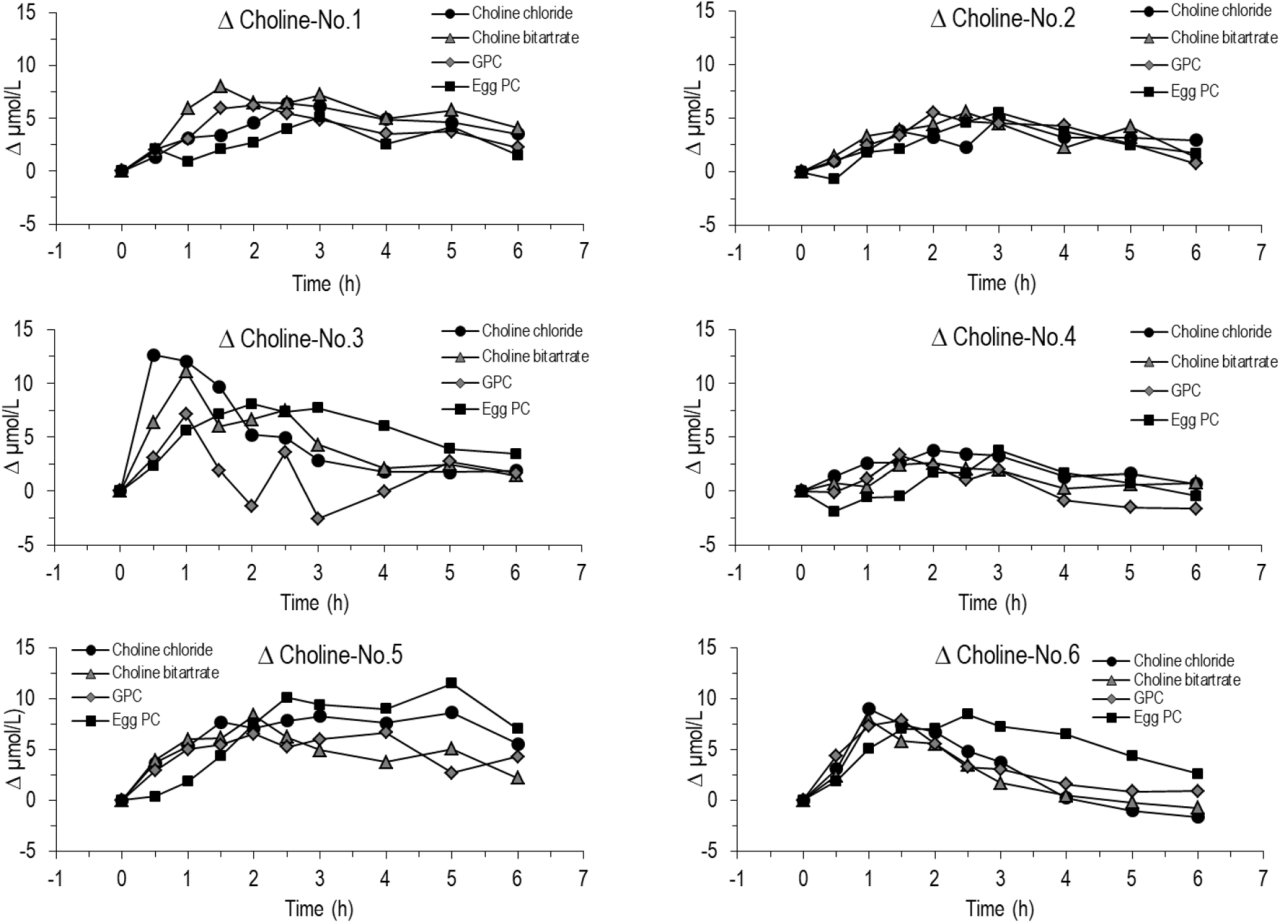
Individual changes of choline concentrations in plasma after the ingestion of choline chloride, choline bitartrate, α-glycerophosphocholine (GPC) or egg-PC

**Fig. 4:**
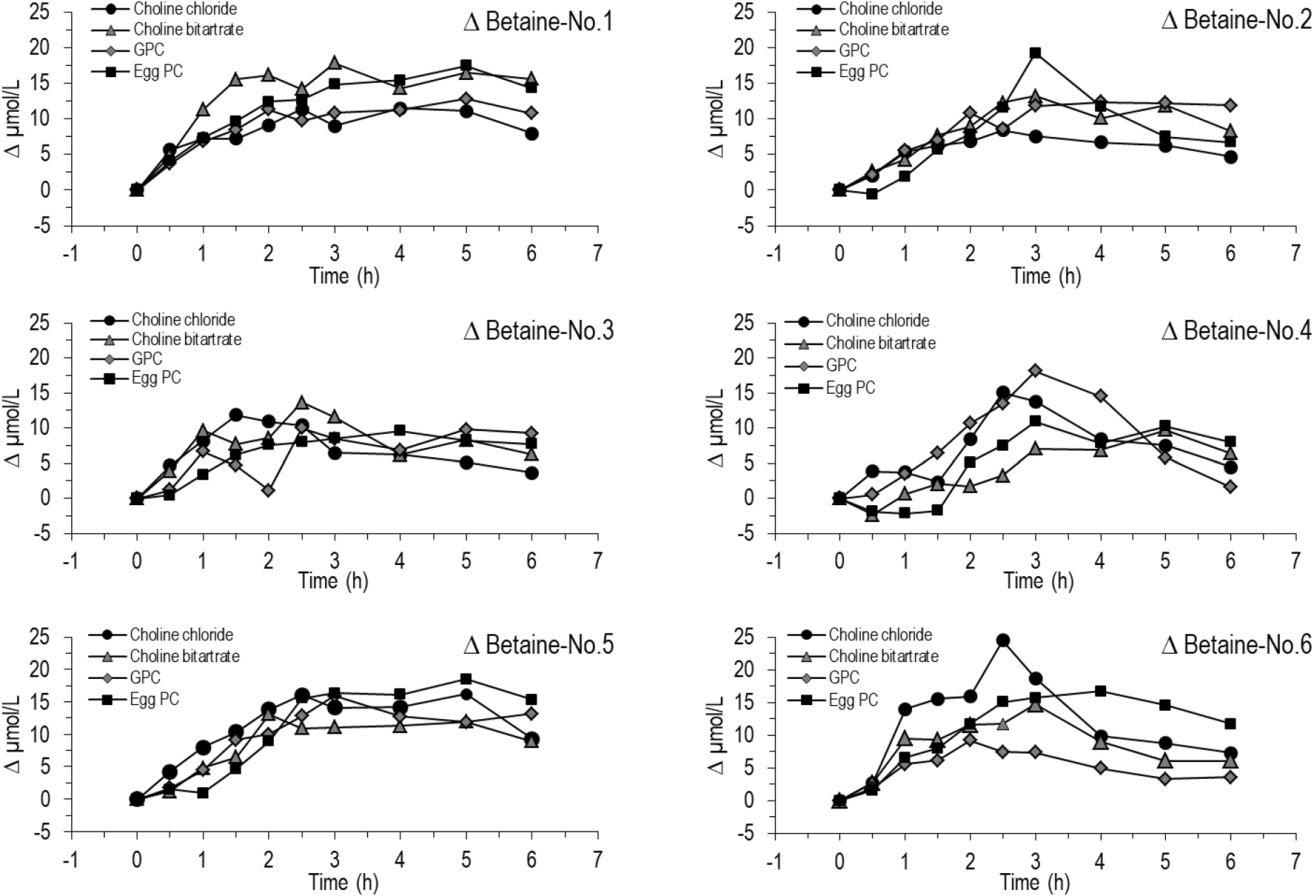
Individual changes of betaine concentrations in plasma after the ingestion of choline chloride, choline bitartrate, α-glycerophosphocholine (GPC) or egg-PC

Individual kinetics of plasma choline and betaine are shown in Figs. 3, 4, respectively. Data demonstrate that the extent and slope of increase differed significantly among individuals. For instance, whereas no. 3 and 6 showed rapid and delayed increases after ingestion of choline chloride and egg-PC, respectively, such differences were less obvious for other individuals (#2 and 4) (Fig. 3). Similarly, the slopes of the increase in plasma betaine differed among individuals (Fig. 4, #1, 3 and 5 vs. #4).

**Fig. 5:**
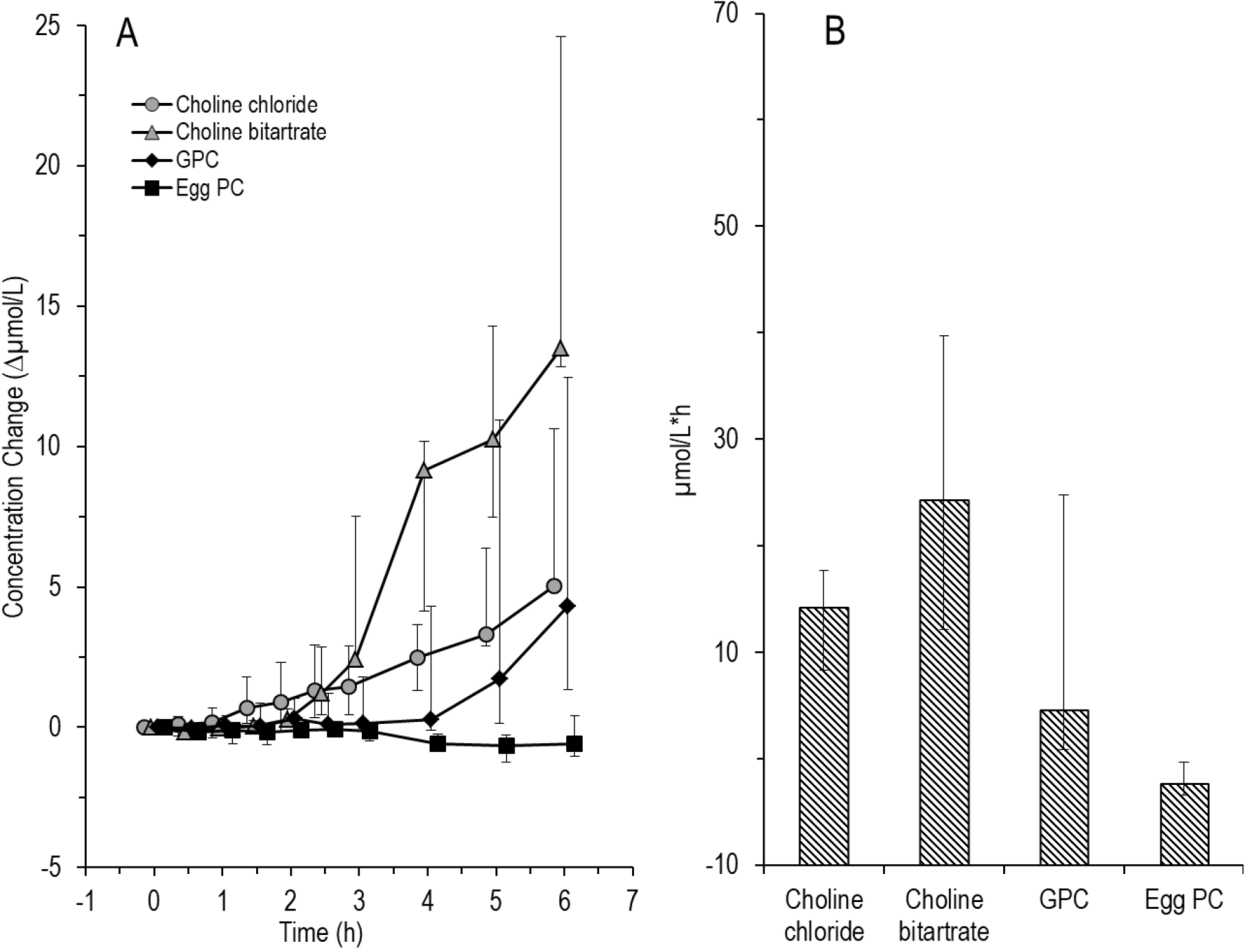
Effect of different choline supplements on change in plasma concentration of TMAO (**A**) and the resulting area under the curve (AUC) (**B**) for TMAO in relation to the respective choline supplement choline components given orally as a single dose of 550 mg choline equivalent together with a test meal. Data are medians and interquartile ranges. *AUC* area under the curve, AUC was calculated with trapezoidal rule

**Fig. 6:**
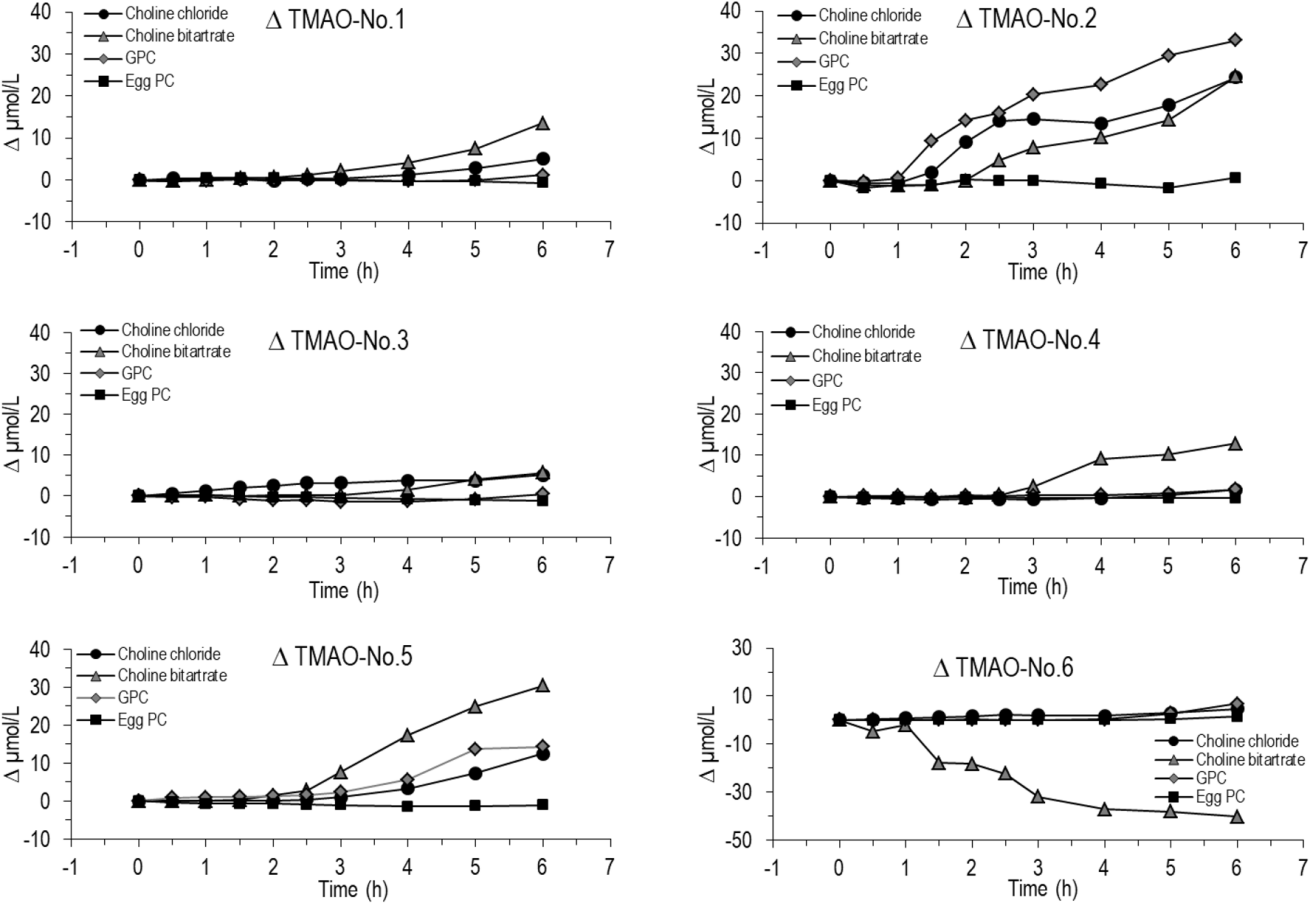
Time course of individual TMAO concentrations in plasma after the ingestion of choline chloride, choline bitartrate, alpha-glycerophosphocholine (GPC) or egg-PC. Due to an exceptional dietary intake of sea food at the evening before, individual No. 6 started with 66.4 µmol/l TMAO at *t* = 0 h in the choline bitartrate experiment, followed by a continuous decrease of TMAO towards the usual range irrespective of the supplement given as depicted herein

Although the areas under the curve for choline did not differ between individual choline supplements (Table 3), they exerted highly differential effects on plasma TMAO concentrations (Fig. 5). All water-soluble components increased plasma TMAO concentrations, whereas egg-PC did not (Fig. 5A), and the AUC value after 6 h was highest for choline bitartrate, but lowest for egg-PC (Fig. 5B). As shown in Fig. 6, no individual showed an increase in plasma TMAO in response to egg-PC, whereas individual responses were highly different after water-soluble choline carriers. Here, subjects #1, 3 and 4 generally showed only an increase by < 10 µmol/l, whereas for #2 and 5, the maximal increase was at 30 µmol/l. Individual # 6 had normal basal TMAO levels of 1.0, 2.0 and 2.0 µmol/l at *t* = 0 in the choline chloride, GPC and egg-PC experiment, and a minor increase (< 5 µmol/l) following supplement administration. However, incidentally and due to dietary fish intake the evening before, TMAO concentration at baseline was 66.4 µmol/l in the choline bitartrate experiment, thereafter decreasing towards the usual range irrespective of the supplement (Fig. 6). At 24 h TMAO had returned to 2.3 µmol/l.

## Discussion

The perception of choline as an essential nutrient, and of a suboptimal intake by a significant part of the overall population has recently emerged, and choline supplementation is frequently suggested [11, 12, 25]. Choline supplementation may be even more important in choline deficiency observed in medical conditions such as premature birth, cystic fibrosis with exocrine pancreatic insufficiency, hepatosteatosis, total parenteral nutrition, and short bowel disease. This suggests a need for such supplementation in the clinical setting, although there are no firm health claims by the European Food Safety Authority (EFSA) [26, 27]. Choline as a body constituent is present in the form of several components, from minor amounts of free choline through its water-soluble organic esters [phosphocholine, cytidine diphosphate-choline (CDP-Choline), GPC, acetylcholine] to the glycero- and sphingolipids phosphatidylcholine (PC) and sphingomyelin (SPH), respectively, as main components [1]. Similarly, free choline is a minor component in most regular food. Fetal life is the only situation where supply is dominated by free choline via the placenta [14]. Infants receive choline with human milk, predominantly containing phosphocholine and GPC, together with PC, lyso-PC, and SPH [28]. In later life, choline is provided by solid food mostly containing PC [29]. Nevertheless, choline salts like choline bitartrate and choline chloride are used in infant formulas, in various clinical trials of choline supplementation, in individual (self) treatment, and in many commercial supplements [30]. Because animal data indicate that the metabolism of free choline, GPC and PC is different [31], we set out to compare GPC, as characteristic for milk, and egg-PC, as characteristic of mixed and vegetarian food, with free choline in the forms of its chloride and bitartrate salts to elucidate potential clinically relevant differences in bioavailability, plasma kinetics, and metabolism. Phosphocholine was not analyzed because it is not available as a food supplement.

Notably, all supplements tested increased choline concentrations by a similar extent. A single dose of 550 mg choline temporarily increased plasma concentrations followed by a rapid decline thereafter so that concentrations after 6 h had nearly reached baseline values for all components. This is surprising as previous data demonstrated a longer-lasting increase in plasma choline in response to egg or soya PC [32]. However, aspects of solubility, i.e., product quality, may contribute to the velocity of PC assimilation, and we only supplemented 4018 mg egg-PC equivalent to 550 mg choline, whereas others used the five-fold amount (20 g) [32].

However, the increase of plasma choline and betaine was delayed after egg-PC compared to other supplements, with a flatter slope of increase and a time to peak concentration of approximately 3 h rather than 1–2 h. This delay is consistent with the mechanism of choline assimilation from PC that requires cleavage of PC at the surface of duodenal lipid micelles to lyso-PC and free fatty acid by pancreatic phospholipase A_2_ IB (sPLA_2_IB) at alkaline small intestinal pH [33]. While choline, according to our data and those of other researchers, is readily assimilated from PC in healthy individuals it must be noted that this process is compromised in exocrine pancreas insufficiency and acidic small intestine pH, as the case in CF patients [26, 34].

All supplements increased plasma betaine concentrations to a similar extent and with similar kinetics, demonstrating that for all supplements a comparable fraction of choline is rapidly degraded to betaine, and that the similar absorption kinetic of choline resulted in a similar kinetic of betaine. Hence, there are apparently no major differences between choline-containing components with respect to their oxidation to betaine. For all substances, the increase in plasma betaine is higher than that of choline, suggesting that feeding the one-carbon pool via betaine is an important aspect of any choline supplementation. Although, for the administration of egg-PC, part of the absorbed lyso-PC is re-acylated to PC and used for the formation of chylomicrons, our data show that there is no difference between PC and the other components with respect to plasma betaine. It is unclear whether this will change after consuming larger amounts of PC or if PC is administered with larger amounts of fat rather than those contained in a butter pretzel.

Due to small sample size, minor differences between the four supplements might not have been detected. Differences in effect size between choline derivatives regarding plasma kinetics or metabolic rates were not known when we planned our experiment. Therefore, the calculation of sample size was based on plasma choline levels of patients with and without choline supplementation.

In this study, we assessed healthy male adults. Women were excluded because of the impact of estrogens on PEMT activity and hence choline metabolism. Transfer of the results of this study to other patient groups such as women and particularly preterm infants should be undertaken with caution. Whereas the kinetics of choline chloride are identical in adult CF patients compared to healthy persons, age, hormones, the microbiome, pancreatic insufficiency and immature digestive enzymes may influence the uptake and metabolism of choline supplements [1; 16], suggesting further studies.

Safety aspects play a major role in nutrition and this is particularly important in patients. Trimethylamine (TMA) is a bacterial degradation product of choline and other trimethylated quaternary ammonium components (betaine, carnitine). Intestinal bacteria degrade choline to TMA which then enters the portal circulation and is oxidized to TMA oxide (TMAO) by flavin-containing monooxygenase 3 (FMO3; EC 1.14.13.148), also called trimethylamine monooxygenase that is expressed in adult but not fetal liver [20]. An increase in TMAO in adult but not preterm patients upon choline chloride supplementation was shown before [3, 26]. TMAO was associated with cardiovascular disease risk, although no firm causality was shown [18]. TMAO returned to baseline values in all groups after 24 h. The importance for long-term health of repeatedly albeit temporarily raised TMAO levels due to long-term choline supplementation is unknown. Notably, egg-PC did not induce an increase in plasma TMAO in any study participant, including those forming high amounts of TMAO upon the ingestion of choline chloride, choline bitartrate, and GPC. Moreover, the ingestion of choline bitartrate exerted the highest increase in three out of five individuals, although individual responses were different. Apparently, the formation of TMA and TMAO depends on individual microbiota, but may be influenced by many factors, like occult inflammation or intestinal transit time. Whereas PC is degraded by colonic microbes, and choline and lyso-PC formed, it is not known whether patients with pancreatic insufficiency and, therefore, fecal choline losses, automatically have increased TMAO levels and whether the supplementation of such patients with PC rather than choline chloride or bitartrate will prevent TMAO formation [3, 34, 35]. It must also be discussed whether egg-PC should be used for supplementation in infants. Concerning the risk of egg protein for allergy development, the supplement must be free of protein, and the risk of TMAO formation must be evaluated. Our previous data have not shown any TMAO in preterm infants following choline chloride supplementation, which is in contrast to adults [3, 26]. While PC is not a major choline carrier in milk-fed term and preterm infants [36], the liver secretes 50% of its PC pool daily into the duodenum, which is cleaved by sPLA_2_IB and neonatal pancreatic lipase-related protein 2 (PLRP2) [33]. Moreover, even in pancreatic insufficiency, part of ingested PC is assimilated [37]. Hence, PC may be a suitable alternative to choline salts to improve choline supply at least in various adult patient groups but potentially also in preterm infants.

## Conclusion

All supplements increase choline levels in a similar way and similarly contribute to the formation of betaine. Assuming that TMAO may have undesirable effects on health, we suggest egg-PC for choline supplementation in adults, because it was the only component not raising TMAO levels. This could be important when for prolonged supplementation. While further studies are necessary, our preliminary data suggested that choline bitartrate may increase TMAO more than choline chloride or GPC. Furthermore, the microbiota may be an important factor in TMAO formation, and its analysis in choline-supplemented patients could be useful.

## Supplementary Information

Below is the link to the electronic supplementary material.

Supplementary file1 (DOCX 15 KB)

## Abbreviations

ACh: Acetylcholine
AI: Adequate intake
AUC: Area under the curve
CF: Cystic fibrosis
EDTA: Ethylenediaminetetraacetate
EFSA: European Food Safety Authority
FMO3: Flavin-containing monooxygenase 3
αGPC: Alpha-glycerophosphocholine
GRAS: Generally Recognized As Safe
NAM: National Academy of Medicine of the USA
PC: Phosphatidylcholine
SIBO: Small intestinal bacterial overgrowth
SPH: Sphingomyelin
TMA: Trimethylamine
TMAO: Trimethylamine oxide
