# Supplementary material for: Differential metabolism of choline supplements in adult volunteers
Source: Eur J Nutr. 2021 Jul 21;61(1):219–30. doi: 10.1007/s00394-021-02637-6 (PMC8783899; doi:10.1007/s00394-021-02637-6)
Supplement: Supplementary file 1 — Supplementary file1 (DOCX 15 KB) [file 394_2021_2637_MOESM1_ESM.docx]

**Supplementary Information – European Journal of Nutrition**

**Differential Metabolism of Choline Supplements in Adult Volunteers**

Böckmann K.A.^1^, Franz A.R.^1,2^, Minarski M.^1,^ Shunova A.^1^, Maiwald C.A. ^1^, Schwarz J. ^1^, Gross M. ^1^, Poets C.F.^1^, Bernhard W.^1^

**Affiliations:** ^1^Department of Neonatology, ^2^Center for Pediatric Clinical Studies

**Address of correspondence**: Katrin Alexandra Böckmann, MD, Department of Neonatology, Faculty of Medicine, Eberhard-Karls-University, Calwer Straße 7, D-72076 Tuebingen, Germany; katrin.boeckmann@med.uni-tuebingen.de; Phone: +49 7071 29 84742

Supplemental Table s1: Area under the curve (choline and betaine), peak time and slope to peak for choline plasma concentrations after the administration of 4 different supplements of 550mg choline equivalent: p-values by Friedman and Wilcoxon signed-rank test

|  | AUC  Choline (0-24h) | AUC  Choline  (0-6h) | AUC  Betaine (0-24h) | AUC  Betaine   (0-6h) | Time to peak  Choline | Slope to peak  Choline |
| --- | --- | --- | --- | --- | --- | --- |
| Friedman | 0.84 | 0.19 | 0.07 | 0.38 | 0.02 | 0.26 |
| Wilcoxon sign-rank test |  | | | | | |
| Choline chloride vs. choline bitartrate | 1 | 0.84 | 0.03 | 0.22 | 0.38 | 1.0 |
| αGPC vs. choline bitartrate | 1 | 0.44 | 0.84 | 0.44 | 0.75 | 0.16 |
| αGPC vs. choline chloride | 1 | 0.44 | 0.03 | 0.22 | 0.34 | 0.84 |
| egg-PC vs. choline bitartrate | 0.44 | 0.44 | 0.56 | 0.84 | 0.06 | 0.03 |
| egg-PC vs. choline chloride | 0.44 | 0.16 | 0.16 | 0.22 | 0.25 | 0.44 |
| egg-PC vs αGPC | 1 | 0.16 | 0.69 | 1.0 | 0.03 | 0.09 |

Supplemental Table s1 shows p-values by Friedman and Wilcoxon signed-rank test comparing AUC (0 until 24 hours and 0 until 6hours) of choline and betaine plasma concentrations and peak time and slope to peak of choline after the intake of the four tested supplements. Abbreviations: AUC= area under the curve, PC: phosphatidylcholine, αGPC: alpha glycerophosphocholine

Supplemental Table s2: choline/betaine ratio of the area under the curve for 24 hours

| AUC 24 choline/betaine | | | |
| --- | --- | --- | --- |
| Friedman p=0.13 | | | |
| Choline chloride | Choline bitartrate | αGPC | egg-PC |
| 0.38 (0.27-0.47) | 0.32 (0.25-0.43) | 0.35 (0.22-0.43) | 0.35 (0.3-0.41) |

Supplemental Table s2 shows the ratio of choline/betaine AUC over 24h after the application of the four supplements. Data are shown as median (25^th^percentile-75^th^ percentile). Abbreviations: AUC= area under the curve, PC: phosphatidylcholine, αGPC: alpha glycerophosphocholine
